# Editorial: Anosognosia in neurological and psychiatric disorders

**DOI:** 10.3389/fneur.2026.1957159

**Published:** 2026-08-24

**Authors:** Lorenzo Pia, Xavier Amador, Daniel Antoniello, Anna Maria Berti, Gabriella Bottini, Eric Andrew Nelson

**Affiliations:** 1 SAMBA (SpAtial, Motor and Bodily Awareness) Research Group, Department of Psychology, University of Turin, Turin, Italy; 2 Department of Psychiatry, The University of Utah, Salt Lake City, UT, United States; 3 Montefiore Medical Center, Albert Einstein College of Medicine, Bronx, NY, United States; 4 Department of Brain and Behavioral Sciences, University of Pavia, Pavia, Italy; 5 Cognitive Neuropsychology Centre, Azienda Socio-Sanitaria Territoriale (ASST) Grande Ospedale Metropolitano Niguarda, Milan, Italy; 6 James J. Peters Veterans Affairs (VA) Medical Center, United States Department of Veterans Affairs, Bronx, NY, United States

**Keywords:** anosognosia, insight, metacognition, neurological disorders, psychiatric disorders, self-awareness

Anosognosia, originally described by Babinski as the lack of awareness of neurological deficits (1), has evolved into a broader concept encompassing impaired awareness of neurological, cognitive, behavioral, and psychiatric symptoms (2). Although first recognized in patients with focal brain lesions, anosognosia is now acknowledged as a common feature of a wide range of neurological and psychiatric disorders, including stroke, traumatic brain injury, Alzheimer's disease and other dementias, Parkinson's disease, aphasic syndromes, schizophrenia, bipolar disorder, and anorexia nervosa (2). Across these conditions, impaired awareness may affect motor, sensory, language, cognitive, behavioral, or psychiatric domains, posing a major challenge for diagnosis, treatment adherence, rehabilitation, and long-term clinical management.

Although our understanding of anosognosia has advanced over the past decades, it remains one of the most challenging phenomena in both neurology and psychiatry. Its clinical heterogeneity suggests that no single mechanism can fully explain impaired awareness. At the same time, advances in neuropsychology, cognitive neuroscience, neuroimaging, and computational modeling have provided new insights into the mechanisms of self-awareness and fostered a more integrated view of anosognosia across disorders.

This Research Topic was conceived to bring together these different perspectives. Through original research articles, reviews, and perspective papers spanning neurological and psychiatric disorders, it provides an updated overview of the field while promoting an interdisciplinary dialogue on impaired self-awareness. The contributions collected here highlight both the diversity of the clinical manifestations of anosognosia and the common mechanisms that may underlie impaired self-awareness across disorders.

A major theme emerging from this Research Topic concerns the mechanisms underlying impaired self-awareness. Several contributions propose complementary frameworks to explain why patients fail to recognize their own deficits. Several contributions propose complementary frameworks to explain why patients fail to recognize their own deficits. Gallingani et al. review the relationship between anosognosia and neuropsychiatric symptoms in neurodegenerative diseases, highlighting the association between impaired awareness and neuropsychiatric symptoms, particularly apathy and depression, and discussing their possible shared neural substrates. This theoretical perspective is further expanded by Seif, who proposes the Third-Person Pathology Heuristic as a novel account of impaired insight in schizophrenia (2), by Sigala et al., who integrate executive dysfunction with predictive and adaptive coding models in acquired brain injury, and by Szalisznyo and Silverstein, who bridge psychiatric and educational metacognition to offer a broader view of impaired self-awareness.

Several contributions examine anosognosia in neurological disorders, emphasizing its heterogeneous clinical expression across different diseases and functional domains. In Alzheimer's disease and related conditions, Coddaire et al. show that reduced awareness of functional competence is associated not only with cognitive impairment, but also with affective and motor dysfunction. Complementing this perspective, Simioni et al. revisit anosognosia for motor deficits after left hemisphere lesions, challenging the traditional view that motor awareness is predominantly a right-hemisphere function. Extending the concept of illness awareness beyond classical neurological syndromes, Song et al. explore its neural correlates in obesity, identifying brain mechanisms that may contribute to reduced awareness of illness. Gu et al. extend the clinical perspective by examining the relationship between optimism, coping strategies, and post-stroke cognitive impairment, highlighting the contribution of psychological factors to recovery after stroke. Together, these studies illustrate the wide clinical spectrum of anosognosia and related disturbances of self-awareness, showing how they may involve different functional domains and arise from partially distinct, yet overlapping, neural and cognitive mechanisms.

The psychiatric dimension of anosognosia is addressed from different but complementary perspectives. Rushani et al. revisit impaired awareness in anorexia nervosa, drawing parallels with anosognosia for hemiplegia and proposing predictive coding and multisensory integration as possible mechanisms underlying persistent denial of illness. Kotzé and Roos focus instead on schizophrenia, discussing the complex relationship between anosognosia and suicide risk and highlighting the ethical implications for future research and clinical practice. Together, these contributions further emphasize that impaired awareness is not restricted to neurological disorders, but represents a clinically relevant dimension across psychiatric conditions as well.

Several contributions also highlight the clinical implications of impaired self-awareness. Eslami-Amirabadi et al. show that anosognosia, together with deficits in Theory of Mind, contributes to caregiver distress across different dementia syndromes, emphasizing the importance of assessing both domains in routine clinical practice. Beyond awareness itself, the studies included in this Research Topic underline how anosognosia influences treatment adherence, rehabilitation, and patient management, reinforcing the need for reliable assessment tools and targeted therapeutic strategies. Finally, Jiang et al. discuss biomarker-informed approaches to post-stroke depression, highlighting how biological stratification may support future personalized interventions.

Taken together, the contributions collected in this Research Topic illustrate the remarkable breadth of contemporary anosognosia research. By integrating clinical observations with cognitive, neuropsychological, neurobiological, and computational approaches, they reinforce the view that impaired self-awareness is a multidimensional phenomenon that transcends traditional diagnostic boundaries. We hope that this Research Topic will stimulate further interdisciplinary research and contribute to the development of more comprehensive theoretical models, improved assessment tools, and more effective clinical interventions for patients with impaired awareness.

## References

[1] Babinski J. Contribution à l‘étude des troubles mentaux dans l'hémiplégie organique cérébrale (anosognosie). Revue Neurol. (1914) 27:845–8.

[2] Prigatano GP. The Study of Anosognosia. Oxford: Oxford University Press (2010).

